# Luminescence from Si-Implanted SiO_2_-Si_3_N_4_ Nano Bi-Layers for Electrophotonic Integrated Si Light Sources

**DOI:** 10.3390/s19040865

**Published:** 2019-02-19

**Authors:** Alfredo A. González-Fernández, Joan Juvert, Mariano Aceves-Mijares, Carlos Domínguez

**Affiliations:** 1 INAOE, Department of Electronics, P.O. Box 51, Puebla 72000, Mexico; aaglz@inaoep.mx; 2 Institut de Microelectrònica de Barcelona, CNM-CSIC, Campus UAB, 08193 Bellaterra, Spain; joan@gollum.cat (J.J.); Carlos.Dominguez@imb-cnm.csic.es (C.D.)

**Keywords:** integrated photonics, electrophotonics, SRO, oxynitride, photoluminescence, XPS

## Abstract

In this paper, we present structural and luminescence studies of silicon-rich silicon oxide (SRO) and SRO-Si${}_{3}$N${}_{4}$ bi-layers for integration in emitter-waveguide pairs that can be used for photonic lab-on-a-chip sensing applications. The results from bi and mono layers are also compared. Two clearly separated emission bands are respectively attributed to a combination of defect and quantum confinement–related emission in the SRO, as well as to defects found in an oxynitride transition zone that forms between the oxide and the nitride films, while ruling out quantum-confinement phenomena in the silicon nitride.

## 1. Introduction

The use of nano-structured Si-based materials has been demonstrated to be a viable alternative for producing fully monolithic electrophotonic systems obtained solely by complementary metal oxide semiconductor (CMOS) techniques [1,2,3]. In particular, light emitters using Si${}_{x}$N${}_{y}$-SiO${}_{x}$ nano-bilayers are promising for several applications, due to the combination of a wide visible spectrum and well-controlled photoluminescence (PL) provided by silicon-rich oxide (SRO) [2], and the possibility of improving efficiency [1], carrier injection due to lower energy barriers [4], and quantum yields [5] provided by silicon nitride and oxynitrides for electroluminescence (EL). On the other hand, planar waveguides (WG) transmitting injected or optically stimulated visible light have shown to be very promising for application in the field of integrated optical sensors and biosensors [6]. Examples of this include integrated Raman sensors [7], sensors based on Mach-Zehnder interferometers [8,9] and spiral resonators [10], and Bragg grating sensors [11]. The possibility of fabricating such photonic systems using CMOS-compatible techniques and materials could enable some important potential advantages, such as reduced costs, high volumes, small size, and easy parallelization to target a variety of mediums [6]. However, most of the current approaches rely on external and non-integrated optical stimulation and detection, which significantly impacts on the integrability and cost of the systems.

We have recently demonstrated an electrophotonic transmitter/receiver consisting of a novel, electrically pumped SRO-nitride visible light-emitting capacitor (LEC) embedded into a planar Si${}_{3}$N${}_{4}$ waveguide surrounded by silicon dioxide cladding [12]. Since the system is fabricated using standard CMOS processes, it was possible to directly integrate a photodiode as the light detector at the output port of the WG, which is a difficult task if using platforms non-standard for integrated electronics, such as silicon-on-insulator (SOI) technology [12]. The use of SRO-nitride bi-layers in light-emitting devices allows for their direct embedding into the WG, thus eliminating alignment and insertion issues since there is no need for external light sources. This monolithic photonic system represents a step forward in the development of fully integrated photonic systems for sensing applications in which localized sections of one or many waveguides can be exposed to enable contact with different mediums to analyze. Depending on the particular analyte characteristics, the light being transmitted is modified along its path to the photodetector, and this results in a change to the delivered electric current. Such variations in photocurrent can be monitored and related to specific characteristics of the analytes.

Nevertheless, when thinking of sensing applications, sufficient control and knowledge of the emission spectra of the light source is of great importance, and while a significant amount of studies have been devoted to the light emission in SRO, there is still much work to do in this regard on dual-layered nitride-SRO systems. Following previous studies demonstrating a close relation between EL and PL in these kind of devices [13], this work presents PL and atomic composition studies on Si${}_{3}$N${}_{4}$-SRO stacks obtained by the implantation of Si ions in the oxide layer of Si${}_{3}$N${}_{4}$-SiO${}_{2}$ bi-layers. The results for dual-film systems and SRO mono-layers are also analyzed and compared. It is shown that the presence of Si${}_{3}$N${}_{4}$ introduces effects in the emission worthy of consideration when designing the systems for specific applications, despite not containing excess Si.

## 2. Materials and Methods

The fabrication of three sets of Si${}_{3}$N${}_{4}$-SRO samples started with the thermal oxidation of p-type crystalline Si wafers at 1100 ${}^{\circ }C$ to obtain uniform, 30 nm-thick SiO${}_{2}$ films. After this, a 30 nm-thick film of Si${}_{3}$N${}_{4}$ was deposited on top of the oxide layers by low-pressure chemical vapor deposition (LPCVD) at 800 ${}^{\circ }C$ using dichlorosilane and ammonia as precursor gasses.

Each set of samples was submitted to a Si${}^{+}$ implantation process performed in two steps to obtain a uniform distribution of excess Si atoms within the oxide layers [13]. Three total implantation doses were used: 1.2 $\times \;10^{16}$ cm${}^{-1}$ (samples *A*), 3 $\times \;10^{16}$ cm${}^{-1}$ (samples *B*), and 1.5 $\times \;10^{16}$ cm${}^{-1}$ (samples *R*). The energies of the implantation processes were tuned according to Stopping and Range of Ions in Matter (SRIM) simulations [14] to obtain the peak of Si ion concentrations in the middle of the oxide film for the sets *A* and *B*, as well as inside the Si substrate in the set *R* in order to obtain SRO films with two different Si concentrations, and a reference set of films with no excess Si in the nitride nor in the oxide film, but which still underwent the implantation to assure that this process by itself did not introduce any kind of emission centers.

All the samples were annealed in a N${}_{2}$ atmosphere for 240 min at 1100 ${}^{\circ }C$ to promote the nucleation of Si excess in the SRO [15]. Finally, the top nitride film was etched from one wafer in each set of samples in order to obtain mono-layers with the same SRO present in the bi-layers. This was done using standard procedures for silicon nitride etching in the CMOS process of the IMB-CNM [16] facilities. This consists of a dip of the wafers in Sioetch to eliminate superficial oxides and oxinitrides, and a further, usually longer, hot H${}_{3}$PO${}_{4}$ bath which is highly selective of silicon nitride [17]. This process is repeated at least twice while supervising the thickness of films using a spectroscopic reflectivity analyzer Nanospec II in order to ensure, as much as possible, the presence of solely the 30 nm bottom oxide, adjusting the etching times of both solutions as needed.

Surface studies of atomic composition in all the samples were performed by X-ray photoelectron spectroscopy (XPS) using a K-Alpha spectrometer with a monochromatic Al source at an energy of $E_{K_{\alpha }}$ = 1486.68 eV. The atomic percentages of Si, O, and N were calculated by integrating the XPS spectra for the energy regions corresponding to the Si2p, O1s, and N1s orbitals, respectively. The presence of other elements was neglected. Depth profiles of atomic contents were obtained by etching the surface of samples using an Ar${}^{+}$ beam sputter in vacuum conditions, and obtaining the XPS spectra for each depth point.

Photoluminescence of the samples was characterized at room temperature and under controlled illumination conditions, pumping the top of the films at 45 ${}^{\circ }$ with UV light at 325 nm and 30 mW. These values are standard and appropriate for PL characterization of similar SRO, as demonstrated previously [2,15,18,19]. The emitted light was collected using an optical fiber oriented normally to the surface and connected to an Ocean Optics QE65000 spectrometer. A long-pass filter and a lens were used to avoid detection of wavelengths below 355 nm and to focus the emitted light on the input of the optical fiber to a maximum detection intensity. The excitation beam was also focused on the surface of samples positioning a lens at the distance of maximum intensity registered by the spectrometer. No significant changes in the shape of PL spectra were observed during the focus adjustments of the pumped light, meaning there was no relevant influence of the power density for the purposes of study here presented. To ensure the same measuring conditions and comparability of spectral results, all the experiments were conducted on an optical table using appropriate optomechanical components to control distances and fix angles equally for all the tests.

## 3. Results and Discussion

**Figure 1:**
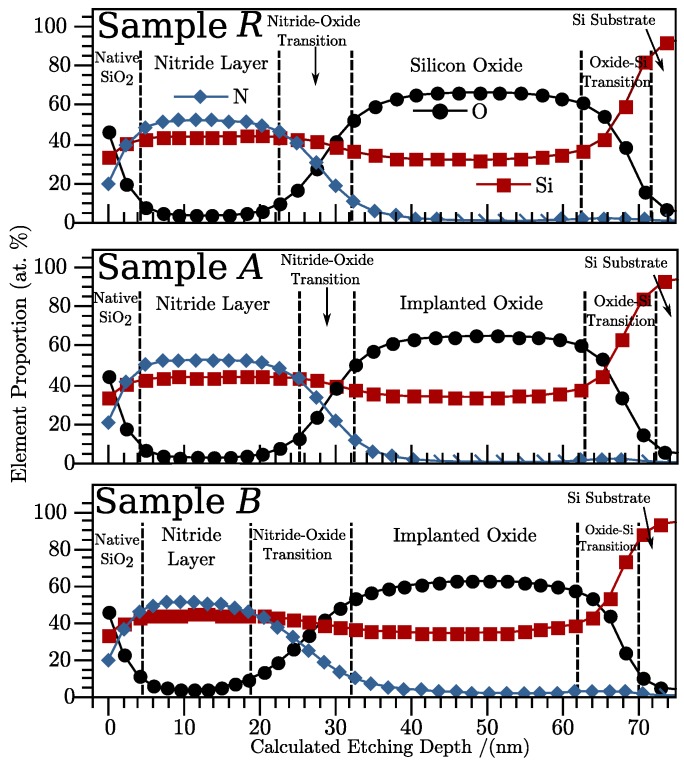
Depth profile of atomic percentage of O, Si, and N as extracted from the areas of the XPS spectra of the O1s, Si2p, and N1s regions, respectively, for bi-layer samples. The points indicate the specific time for which the spectra were obtained, whereas the lines are only for eye guidance.

Figure 1 presents the results for the bi-layer samples of depth profiles of atomic percentages of O, Si, and N. The profiles show that the multi-layer systems were formed by films of Si${}_{3}$N${}_{4}$, SiOx, the Si substrate, and transition regions in between the different materials. The samples present noticeable differences in the widths of transition zones from nitride to oxide, here defined as the region in which there are contents of N and O which are simultaneously higher than 10 at. %. The analysis of the Si2p bands from the XPS results in regard to these depth points showed significant counts for binding energies from 101.9 eV to 103.4 eV, clearly indicating the presence of N–Si–O bonds, meaning this is an oxynitride film [5,20]. The bi-layer sample *B* presents a nitride-oxide transition zone at least 42% wider than the bi-layer sample *A*. In contrast, the thickness of the oxide regions do not change significantly, meaning that the increment in width of the transition region resulted from a decrease of the nitride layer width. The augmentation of the transition zone width matches the increase of the implantation dose. One possible explanation for this is that the damage caused to the atomic structure of the films and a subsequent restructuring with thermal annealing facilitated the interchange of elements between the two layers in the interface region.

Table 1 summarizes the average atomic percentage of Si, N, and O in the oxide films considering the data in the homogeneous region of the SRO films. The homogeneous region is defined as the zone in which the Si contents’ difference between a depth point and the subsequent is lower than 2.5%. There are insignificant differences between these results when comparing SRO in single films to SRO in bi-layers. Results from a thermally grown SiO${}_{2}$ film with no implantation are also shown for comparison (the sample is labeled “Pilot SiO${}_{2}$” in Table 1). The band corresponding to the Si2p orbital energy range was studied in more detail to establish the proportion of electrons corresponding to the Si${}^{0}$ state—that is, those coming from Si to Si atomic bonds. This proportion is also shown in Table 1, and is usually related to the presence of Si agglomerates in the material, if there is any [21].

The results indicate no Si excess in the *R* sample, and detailed study of its Si2p band showed proportions of the Si oxidation states corresponding to Si${}^{4+}$ and Si${}^{0}$ around 98.25% and 0.21%, respectively. These were practically the same as those obtained in the pilot SiO${}_{2}$, confirming that the oxide layer in samples *R* cannot be considered SRO, and it is much closer to SiO${}_{2}$, despite having been subjected to the implantation process.

Table 2 shows the same analysis for the nitride films, averaging the values obtained at the homogeneous zones of the samples (typically between 5 nm and 15 nm from the surface). No Si excess was observed in any of the Nitride films in the samples.

**Figure 2:**
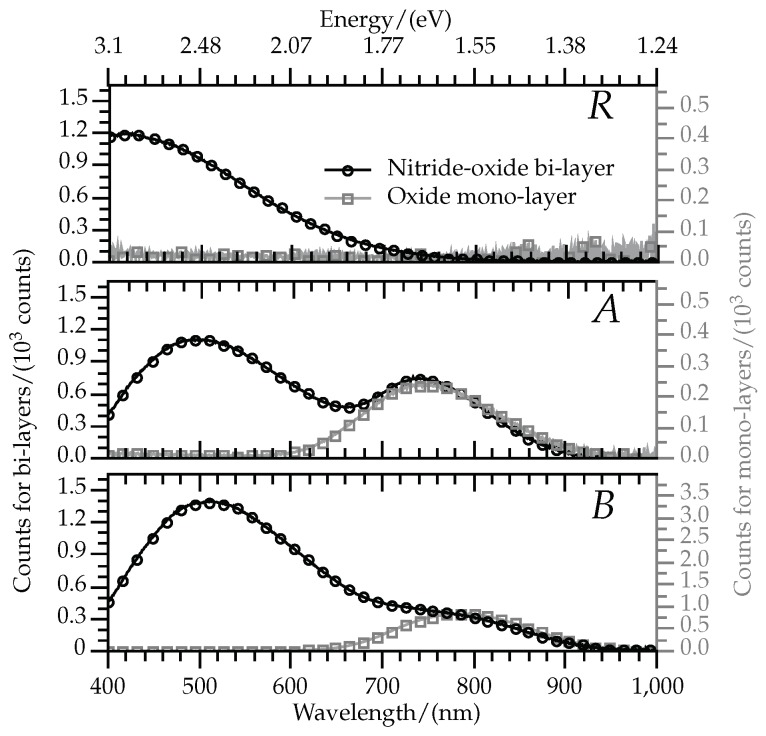
PL spectra of the bi-layer (circles) and their corresponding mono-layer samples (squares). The left y-axis presents the scale for the results of the bi-layers, and the right one shows that of the mono-layers.Photoluminescence (PL) Spectra of bi-layer and mono-layer samples

Figure 2 shows the PL spectra for all the samples, superimposing the results of single oxide films to their corresponding bi-layers. Analyses of absolute intensities are left for a better-suited study, but the experiments here reported allow for a very useful comparison of spectral characteristics, which show a clear match for wavelengths longer than 650 nm between the mono- and bi-layers.

The bi-layered films with Si excess in the oxide film showed two local maximums in PL spectra, while the single SRO films presented only one peak. Additionally, the single oxide film with no Si excess (mono-layered *R* sample) presented no emission, while its corresponding sample with nitride and oxide did show intense PL in the wavelengths below 650 nm.

Since the nitride etching process was designed to avoid significant modifications of the single-standing oxide films after nitride removal, the same PL emission from that particular material in both mono- and bi-layer sets is expected. This is consistent with the match between both PL spectra in mono and bi-layers for wavelengths longer than 650 nm. The resemblance of XPS results from the sample *R* and from the thermal SiO${}_{2}$ pilot is also consistent with the absence of emission at such wavelengths in those samples.

This is supported by previous reports on PL from SRO, which has been extensively studied in the past [2,3,15,18,19,21]. According to these, the bands with longer wavelengths is consistent with a combination of defects and QC-related emission. The defect-related center was likely caused by interface states due to Si=O bonds [18]. These are either present in the nanoparticle-oxide interface (as expected in samples *B* according to the XPS results), or in a pre-formation stage of nanoparticles when not formed in the SRO (as expected in samples *A*).

On the other hand, emission with wavelengths shorter than 650 nm can only be caused by the presence of a nitride film, as it is only observed in the bi-layers. Similar SRO-nitride lattices have shown PL with a distinction between radiation caused by each material, having attributed the nitride-related emission to an excess of Si into it [4]. However, the samples analyzed here show no significant silicon excess in the homogeneous zone of the nitride, as shown in Table 2 and in Figure 1. Table 2 and Figure 1 also show how all samples present very similar composition in the first 15 nm of nitride, despite receiving different Si-implantation doses. Absence of Si excess in the nitride means that the emission related to its presence is not caused by Si agglomerates. This is supported by reports of similar luminescence in silicon-rich silicon nitride (SRN) ruling out phenomena related to quantum confinement as the cause of emission [22,23]. Furthermore, PL tests performed on single-standing nitride films and non-annealed bi-layers did not show detectable PL. All this indicates that the light emission in the blue side of the spectrum originates in the transition region between nitride and oxide.

Moreover, this band matches a combination of different relative contributions by a variety of well-known centers observed in silicon nitrides and oxynitrides [5,24,25,26,27]. Emission within the same wavelengths is likely to be caused by the contribution of emission from centers introduced by nitrogen dangling bonds in SiN${}_{x}$O${}_{y}$ films [26,27] (this is the only significant contribution to luminescence in sample *R*), transitions between the silicon nitride conduction band and defect levels of the type ≡Si${}^{0}$ [24], and defect levels of either the type ≡Si${}^{-}$ [27] or ≡Si${}^{0}$ [25].

**Figure 3:**
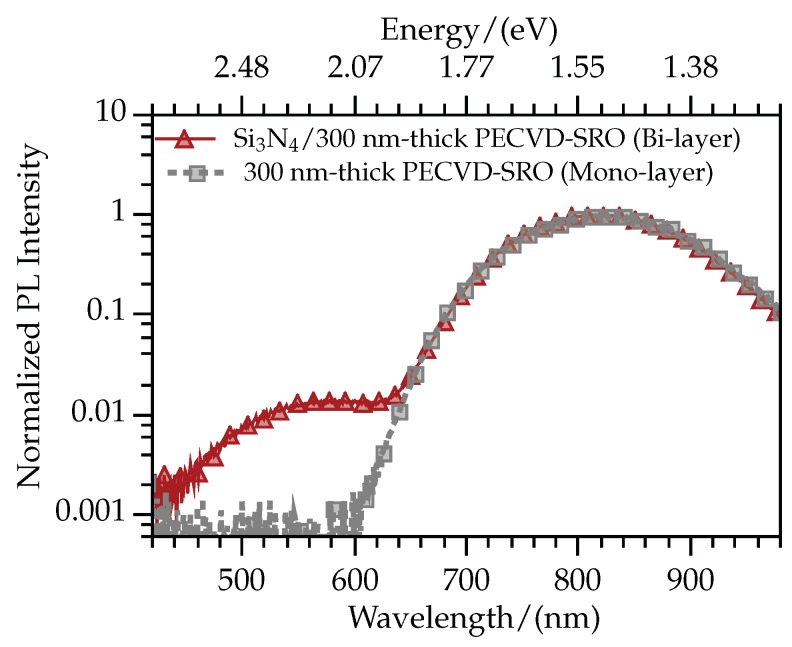
Normalized photoluminescence spectra of mono and bi-layered samples with 300 nm-thick PECVD-SRO. Note that the intensity axis is in logarithmic scale.

Thicker films of well-known SRO fabricated by plasma-enhanced chemical vapor deposition (PECVD-SRO) were used to further confirm that the higher and lower energy bands are respectively caused by the presence of nitride and SRO. In this case, the PECVD-SRO was 300 nm-thick, and fabricated with intrinsic Si excess using the parameters detailed in [21]. After the PECVD-SRO deposition and annealing, the following steps were the same for the fabrication of *R* samples: a 30 nm-thick Si${}_{3}$N${}_{4}$ deposition, Si implantation process, and nitride elimination for the mono-layer. Figure 3 shows the PL results for such samples. Note that in this case, the PL intensity is presented in logarithmic scale as the thickness of PECVD-SRO is one order of magnitude larger than that of nitride. In linear scale (not shown), the emission below 600 nm is not apparent. However, zooming or using the proper scale, the expected two bands in the bi-layered sample can be identified, finding again a clear distinction between emission caused by PECVD-SRO (present in mono and bi-layered samples), and emission caused by nitride (present only in the bi-layered sample). The higher intensity of the emission with longer wavelengths is consistent with the greater thickness of the PECVD-SRO.

## 4. Conclusions

Atomic and photoluminescent characteristics of nano-scale bi-layered SRO–Si${}_{3}$N${}_{4}$ and mono layered SRO films were analyzed. Different Si concentrations were introduced and measured in the SRO films. For each bi-layer film, there was a mono-layer counterpart with the same Si concentration in the SRO. As intended during the fabrication process design, no Si excess was observed in the nitride films. Bi-layers presented a broad PL spectrum with wavelengths from below 400 nm to 920 nm, with two well-defined peaks corresponding to two clearly separated bands. No emission was observed in single-standing nitride films or non-annealed bi-layers. The single SRO films showed only one peak, which presented the same characteristics of bands at longer wavelengths observed in the corresponding bi-layer. Both in annealed mono and bi-layers, the luminescence between 650 nm and 920 nm originated in the SRO films, and was attributed to a combination of quantum confinement-related phenomena, and the existence of radiative defects. The emission with wavelengths below 650 nm was only present in the annealed bi-layers, and found to be originated in a transition oxynitride zone promoted by and dependent on the implantation process. The thickness of these interlayer materials was comparable to those of the SRO and nitride films. Detailed analyses of the PL spectra originated in the oxynitride showed that it was caused by a combination of four previously known radiative centers introduced by N dangling bonds and O vacancies. The results indicate that the presence of a nitride film on top of SRO can significantly modify the emission spectra if the SRO is obtained using ion implantation, even if there is no Si excess in the nitride layer. This is promising for the design of integrated optical sensors in a nitride wave guide scheme, as the range of emission wavelengths can be tuned and comprises most of the visible spectrum, which is quite suitable for a variety of sensing and biosensing applications.

## Figures and Tables

**Table 1:** Elemental atomic percentages and proportion of the total Si2p spectra area by the Si${}^{0}$ oxidation states in the implanted oxide and SiO${}_{2}$ films of bi-layer samples. The levels of N in the thermally grown SiO${}_{2}$ (sample Pilot SiO${}_{2}$) were below minimum reading values.

Sample	Element Contents in Oxide/(at. %)			Si${}^{0}$ Prop. in
	Si	N	O	SRO/(%)
Pilot SiO${}_{2}$	32.99 ± 0.18	-	67.01 ± 0.18	0.20 ± 0.02
*R*	32.70 ± 0.20	1.65 ± 0.12	65.65 ± 0.18	0.21 ± 0.02
*A*	33.78 ± 0.23	0.84 ± 0.19	65.39 ± 0.21	0.86 ± 0.09
*B*	34.57 ± 0.09	2.25 ± 0.47	63.18 ± 0.44	0.59 ± 0.06

**Table 2:** Elemental atomic percentages in the nitride layers of bi-layer samples. The results of a pilot single Si${}_{3}$N${}_{4}$ film deposited by low-pressure chemical vapor deposition (LPCVD) are presented for comparative purposes.

Sample	Element Contents in Nitride Layer/(at. %)		
	Si	N	O
Pilot Si${}_{3}$N${}_{4}$	43.41 ± 0.35	52.89 ± 0.40	3.70 ± 0.75
*R*	43.97 ± 0.10	52.01 ± 0.38	4.02 ± 0.40
*A*	44.03 ± 0.13	52.71 ± 0.29	3.25 ± 0.38
*B*	44.35 ± 0.37	50.66 ± 0.63	4.99 ± 0.96
